# Hypertrophic cauda equina syndrome in mycobacterial meningoradiculitis

**DOI:** 10.1007/s10072-026-09176-0

**Published:** 2026-06-24

**Authors:** Rocco Ricciardi, Laura Fionda, Francesca Tari Capone, Miriam Lichtner, Luca Leonardi

**Affiliations:** 1 Department of Neuroscience, Mental Health and Sensory Organs (NESMOS), Sapienza University of Rome, Rome, 00189 Italy; 2 Neurology Unit, Neuromuscular and Rare Disease Centre, Sant’Andrea Hospital, Rome, 00189 Italy; 3 Neuroradiology Unit, Sant’Andrea Hospital, Rome, 00189 Italy; 4 Infectious Disease Unit, Sant’Andrea Hospital, Rome, 00189 Italy

A 36-year-old man presented to the emergency department with progressive gait disturbance and left facial paralysis. He also reported a one-month history of sensory disturbances and paresthesias in the lower limbs, followed by a more recent onset of lower limb weakness.

Neurological examination revealed an asymmetric distal-predominant motor and sensory deficit in the lower limbs, with absent deep tendon reflexes, left peripheral facial nerve palsy, dysphagia, and dysphonia. The upper limbs were neurologically intact.

**Fig. 1:**
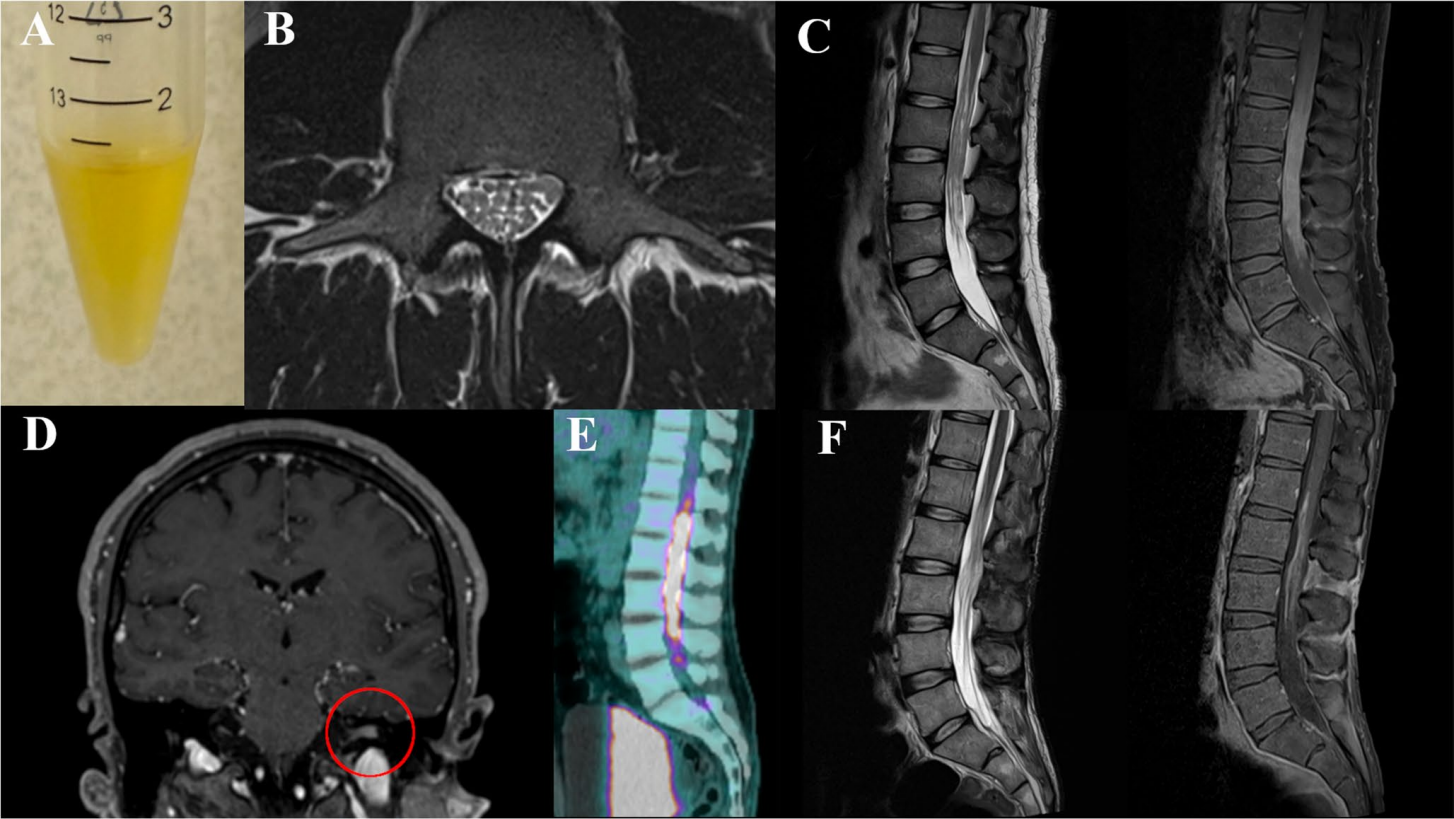
**A** Cerebrospinal fluid (CSF) appearance showing marked xanthochromia; **B** Pre-treatment axial T2-weighted MRI images; **C** Pre-treatment sagittal T2-weighted and contrast-enhanced fat-suppressed Dixon MRI images demonstrating marked thickening and clumping of the cauda equina nerve roots with associated enhancement; **D** Coronal reconstruction of a post-contrast three-dimensional T1-weighted fat-suppressed gradient-echo MRI sequence showing thickening and enhancement of the left facial–vestibulocochlear nerve complex; **E** 18F-FDG PET/CT demonstrating significant radiotracer uptake in the spinal cord; **F** Follow-up MRI performed after 3 months of therapy showing a significant reduction in the volume of the cauda equina nerve roots with decreased contrast enhancement

Cerebrospinal fluid (CSF) analysis revealed marked xanthochromia (Fig. 1A), pleocytosis (1,521 cells/mm³; 97% lymphocytes), hypoglycorrhachia (5 mg/dL), and hyperproteinorrhachia (3,310 mg/dL). A multiplex PCR panel (FilmArray) for neurotropic pathogens was negative.

Contrast-enhanced MRI (Fig. 1B-C) showed involvement of the cauda equina and the left seventh cranial nerve (Fig. 1D), with hypertrophy of the nerve roots and contrast enhancement.

18 F-FDG PET/CT demonstrated intense uptake in the cauda equina (Fig. 1E) and in the left facial–acoustic nerve complex, with no evidence of other pathological uptake. A comprehensive onconeural antibody panel was tested in both serum and CSF, and a total-body CT scan was performed; all results were negative.

As part of the infectious workup, interferon-gamma release assays and culture-based tests on CSF were negative, as were repeated PCR assays for Mycobacterium tuberculosis.

Over the following 10 days, despite broad-spectrum antibiotic therapy, the clinical condition progressed to complete cauda equina syndrome with areflexic paraplegia and neurogenic bladder, associated with bilateral cranial multineuropathy. No clinical response was observed following treatment with high-dose corticosteroids and intravenous immunoglobulins.

In light of progressive clinical deterioration and lack of response to prior treatments, empirical antimycobacterial therapy was initiated based on neuroimaging findings and CSF cytochemical profile, despite negative microbiological tests.

Over the following weeks, CSF parameters and MRI findings (Fig. 1F) [1] gradually improved, with associated clinical stabilization. Gradual clinical improvement was observed only after five months of treatment, with regression of cranial nerve deficits and partial recovery of lower limb function.

Microbiological confirmation of mycobacterial infection was obtained only at a later stage: both adenosine deaminase (ADA) testing [2] and in vitro lymphocyte stimulation assays were positive in blood and CSF samples. Notably, in vitro exposure to TB1 and TB2 antigens elicited a TNF-alpha–mediated lymphocyte response rather than an interferon-gamma–mediated response, which may explain the negative interferon-gamma release assay results.

The decision to start treatment before etiological confirmation was supported by the characteristic neuroimaging and CSF features. This early therapeutic approach likely halted disease progression and contributed to a more favorable outcome, despite initially negative microbiological investigations.
